# A miniaturized spectrometer for NMR relaxometry under extreme conditions

**DOI:** 10.1038/s41598-019-47634-2

**Published:** 2019-08-01

**Authors:** Yiqiao Tang, David McCowan, Yi-Qiao Song

**Affiliations:** Schlumberger-Doll Research, Cambridge, MA 02139 USA

**Keywords:** Physics, Engineering

## Abstract

With the advent of integrated electronics, microfabrication and novel chemistry, NMR (Nuclear Magnetic Resonance) methods, embodied in miniaturized spectrometers, have found profound uses in recent years that are beyond their conventional niche. In this work, we extend NMR relaxometry on a minute sample below 20 *μ*L to challenging environment of 150 °C in temperature and 900 bar in pressure. Combined with a single-board NMR spectrometer, we further demonstrate multidimensional NMR relaxometries capable of resolving compositions of complex fluids. The confluence of HTHP (high-pressure high-temperature) capability, minimal sample volume, and reduced sensor envelop and power budget creates a new class of mobile NMR platforms, bringing the powerful analytical toolkit in a miniaturized footprint to extreme operating conditions.

## Introduction

NMR, considered as one of the most potent analytical methods, traditionally demands superconducting magnets^1^, sizable electronics, and intricate probe and antenna placements^2,3^. Only recently, owing to the advancement in permanent-magnet design^4,5^, electronics integration^6,7^, and antenna miniaturization^8,9^, portable NMR systems^10^ have emerged as a viable surrogate. Thanks to the reductions in footprint, maintenance needs and price tag, the miniaturized sensor assemblies have extended their uses beyond conventional NMR laboratories to a broad range of “field” applications, including point-of-care medical diagnostics^11,12^, flow metering^13,14^, fluid authentication^15,16^, porous material characterization^17,18^, and artefact preservation^19,20^.

Constrained by the current design of permanent magnet, many miniaturized systems focus on measuring NMR relaxations^11,21,22^ that originate from interactions of the spin system with its molecular environment^23^. A major deficiency of these small systems, however, is their limit on operating environment to ambient conditions. While applications of NMR abound at elevated pressure and/or temperature settings, such as in subsurface explorations^24^, polymer dynamics^25,26^, catalysis^27^, hydrogen storage^28^, and gas adsorption in nanoporous materials^29,30^, those measurements always demand complex mechanical designs, large space and numerous pieces of equipment. In this context, miniaturized and integrated NMR platforms with HTHP (high-temperature, high-pressure) capability could further the technique to a substantially broadened usage.

A number of challenges arise when conduct NMR relaxometry within a minimal envelop under HTHP conditions. For example, the reduced volume leads to degraded SNR (signal-to-noise ratio), which further deteriorates at high temperatures. The small volume also corresponds to an increased surface-to-volume ratio of the sample contained in a capillary; accordingly, surface relaxations from the walls of sample tubes, incurred by paramagnetic impurities^31^ and geometrical defects^32,33^, need to be meticulously characterized and rectified. At elevated temperatures, sample relaxation times tend to increase owing to the accelerated molecular motion, demanding systems of superior stability that maintain phase coherence through an extended experimental duration. Finally, the HTHP requirements put further constraints on the selection of capillary materials, the probe design, and associated electronics.

We hereby report the development of a miniaturized NMR platform that is capable of carrying out high-quality relaxation measurements (both *T*_1_ and *T*_2_) within a micro-autoclave up to 150 °C and 900 bar hydraulic pressure. In particular, *T*_2_ ~ 10 s is obtained from a pressurized water sample at 150 °C and 137 bar, with a detection volume below 20 *μ*L. We further show an integrated system, based on an NMR-ASIC (Application Specific Integrated Circuitry) chipset^6^ and a pressure-compensated NMR probe, that can perform a wide range of NMR measurements on a rich diversity of complex fluids.

## Capillary Tube

Functions of the capillary tube are two-fold: it contains the sample under study and provides the mechanical structure to which the RF antenna is attached. Accordingly, the capillary material needs to be non-magnetic, non-conductive, ideally of zero magnetic susceptibility, easy to machine, and can withstand HTHP conditions. The tube is also required to have an ultra-smooth inner wall that is in direct contact with the sample, minimizing surface-induced mobility restriction of fluid molecules.

**Figure 1:**
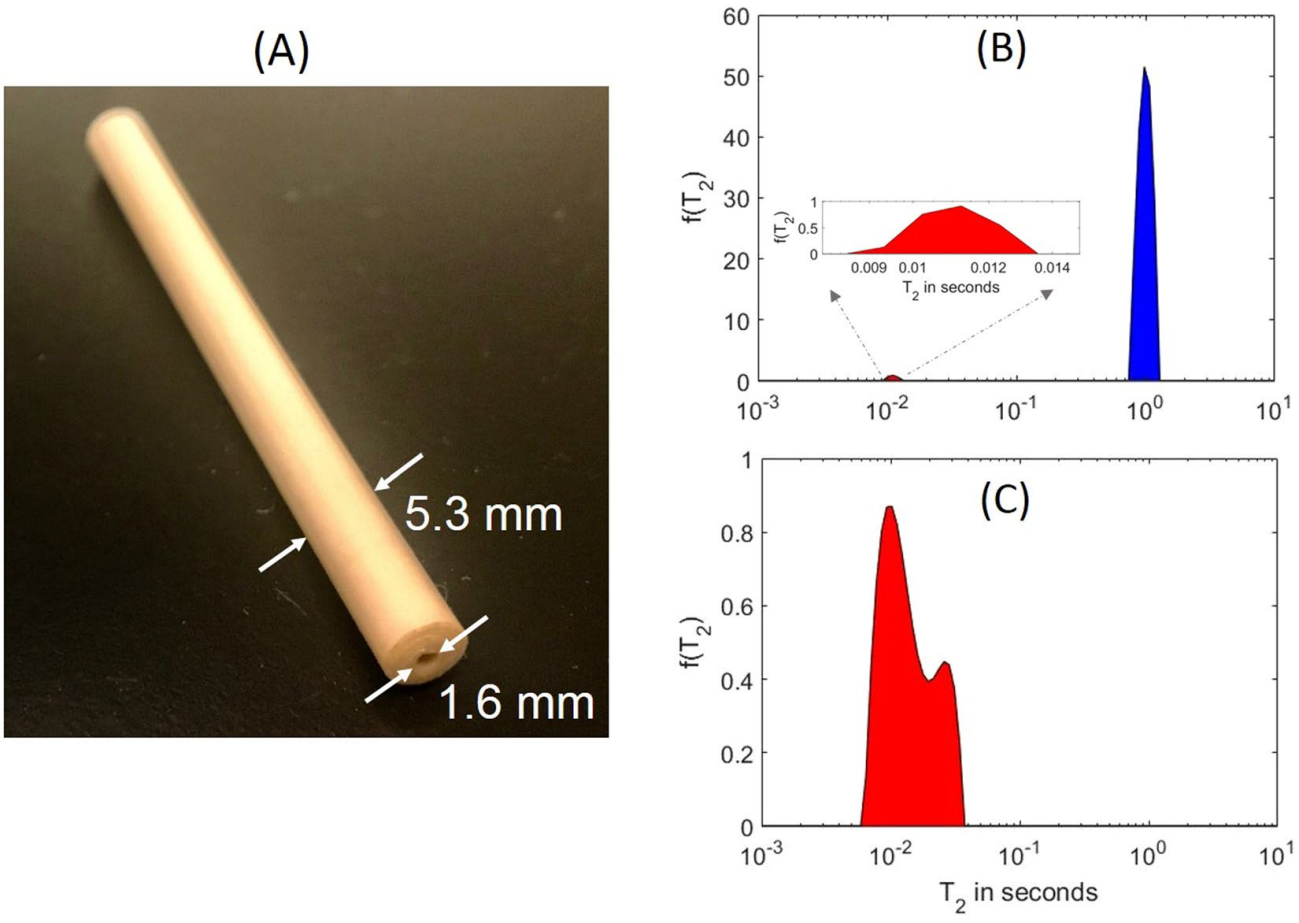
Characterization of the extruded PEEK capillary. (**A**) A photo of the as-received extruded PEEK tube; (**B**) The *T*_2_ spectrum of dodecane contained in the PEEK tube, with the inset showing the zoom-in of the fast-relaxation component; (**C**) The *T*_2_ spectrum of a dried PEEK tube.

For the HTHP sensor, we chose PEEK (polyether ether ketone) as the capillary material thanks to its high melting point (335 °C), relatively high tensile strength, and exceptional chemical and hydrolysis resistance. To avoid contaminating the capillary inner wall, neither physically nor chemically, we used extruded PEEK tubes by Zeus Inc, with an OD of 5.3 mm and an ID of 1.6 mm, as the raw material shown in Fig. 1A.

To evaluate contributions to the measured signal from protons in the capillary material, we wound a solenoidal coil of 1 cm in length over the PEEK tube. We then placed the dodecane-filled capillary tube inside a Halbach-array magnet of 0.54 T *B*_0_ and performed NMR CPMG measurements^34,35^ with a Kea2 spectrometer by Magritek. Subsequently, a fast Laplace inversion (FLI) routine^36^ was applied to invert time-domain signals to distributions of *T*_2_ relaxation times.

As shown in Fig. 1B, a sharp peak was observed at *T*_2_ ~ 1 s, corresponding to relaxation times of the bulk fluid under ambient conditions (T = 21 °C, P = 1 bar), accompanied by a small peak at *T*_2_ = 10 ms. To clarify its genesis, we conducted a control experiment on the same but completely dried tube, as shown in Fig. 1C. Since the fast-relaxation component of equal amplitude persisted, we assigned its origin to the PEEK matrix.

NMR relaxometry is a volumetric measurement, so the ratio of integrals under two spectral peaks represents relative signal strength of their respective origins. In this case, the PEEK signal was 2% of the fluid peak. Furthermore, the modified capillary installed in the NMR probe had an OD of 3.2 mm in the antenna section, corresponding to a further 63.5% reduction of the polymer volume from the as-received tubes. We later show that the matrix contribution was negligible in an assembled NMR probe.

When the operating temperature rises above the PEEK’s glass transition temperature, 143 °C, stress accumulated during the manufacturing process will be released, resulting in a 2% shortening of the tube length. To avoid the deformation, we annealed the polymers at 200 °C per the procedure in Table 1. The annealed capillaries were further machined to fit in an HTHP NMR probe.

**Table 1:** PEEK annealing procedure.

Start temp (°C)	End temp (°C)	Ramp rate (°C/min)	Duration (hrs)
20	150	0.1	21.7
150	150	0	3
150	200	0.1	8.3
200	200	0	3
200	20	−0.1	30

## HTHP NMR Probe

**Figure 2:**
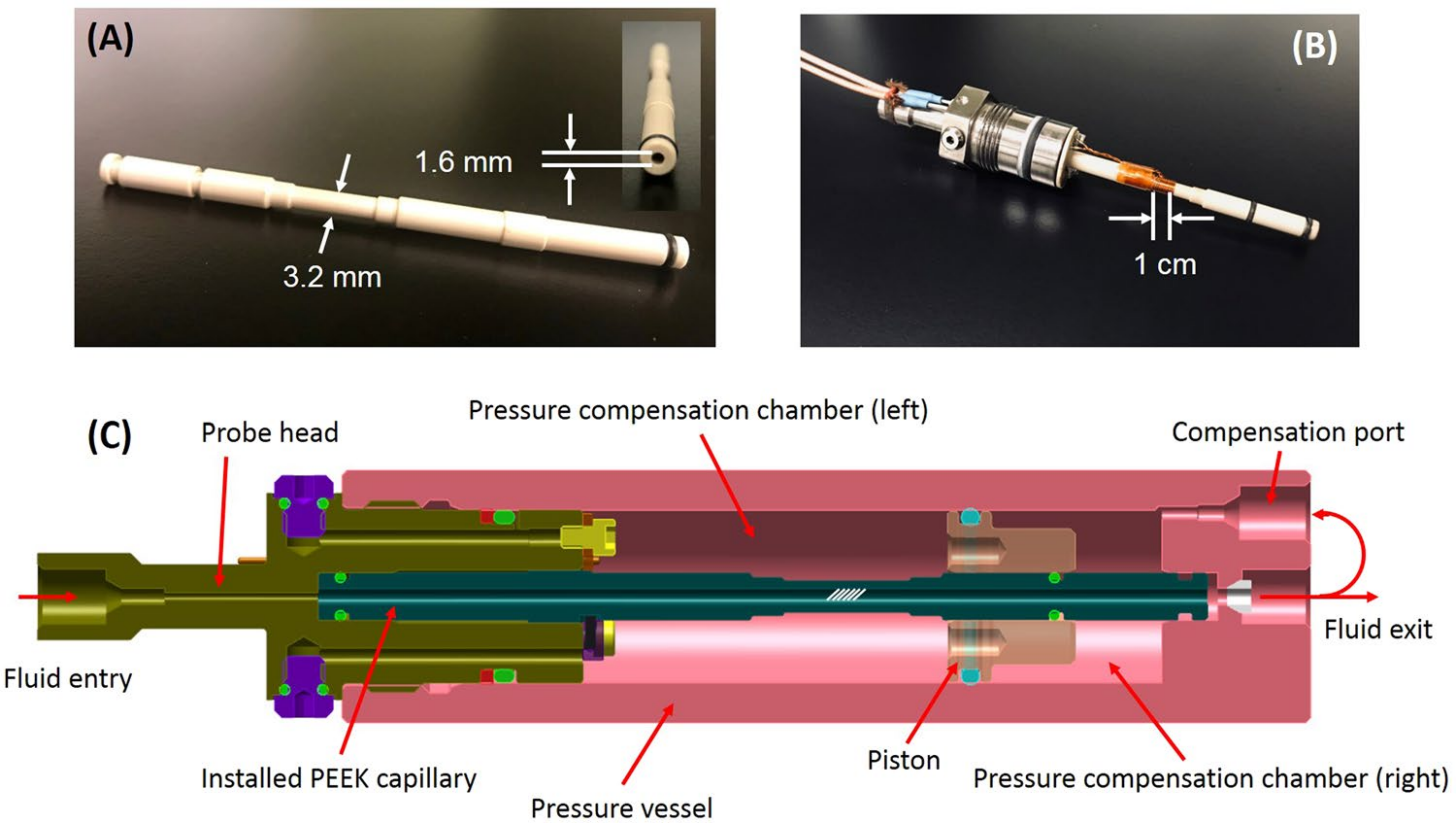
The HTHP NMR probe. (**A**) The machined PEEK capillary, both side view and front view (inset); (**B**) The installed PEEK capillary on the probe head; (**C**) A schematic cross section of the HTHP probe. To equalize pressures across the capillary wall, the space in between of the capillary outer wall and the pressure vessel is filled with fluids of equal pressure. The annular space is further partitioned into two chambers (left and right) by a PEEK piston. To avoid interference with signals from the sampled fluids, the pressure-compensation fluids in the left chamber are fluorinated hydrocarbons. In contrast, the fluids in the right chamber come from the capillary, which enter the space through the compensation port. When the operating condition varies, the piston moves so as to maintain pressure equilibrium across the capillary wall.

To minimize the differential pressure across the thin wall of PEEK capillaries, we designed a pressure-compensation fixture shown in Fig. 2. The idea was to immerse the capillary in a bath of fluorinated hydrocarbons (Fluorinert by 3 M) that didn’t contain any protons. Fluorinert acted as a pressure-compensation fluid that was separated from the sample fluid by a movable piston, so that the two fluids had no substance exchange while maintaining pressure communication. When the operating condition varied, the piston moved to equalize hydraulic pressures across the capillary wall. All the fluids were contained in a vessel made of titanium alloy that could hold a differential pressure up to 900 bar.

To achieve pressure management while meeting the spectrometer specifications, we further machined the PEEK tube that included three O-ring grooves and indentations, as shown in Fig. 2A. During the machining process, the inner wall of the tube was supported by a through-hole gauge pin, and was therefore preserved in its pristine condition. The section of antenna placement had an OD of 3.2 mm, an ID of 1.6 mm, and an effective sample volume of 17 *μ*L.

Subsequently, a solenoidal coil of 11 turns of 28 AWG wires, with inductance *L*_*c*_ of 285 nH and resistance *R*_*c*_ of 1.35 ohm measured at the Larmor frequency of *f*_*L*_ ~ 23 MHz, was wound around the middle section of the machined tube. The quality factor of the coil was 2*πf*_*L*_*L*_*c*_/*R*_*c*_ = 30. After assembling the probe, samples were introduced via the fluid entry while NMR measurements were performed within the shaded section of the capillary in Fig. 2C.

## Magnet, Electronics, and The System

All the experiments were performed with a samarium-cobalt Halbach magnet, designed and manufactured by One Resonance Sensors, LLC. The magnet had a construct of hollow cylinder, with an ID of 25.4 mm, an OD of 70 mm, and a length of 76 mm. A 17 *μ*L cylindrical measurement volume of ca. 100 ppm field homogeneity, with a diameter of 1.5 mm and length of 1 cm, was created at the center of the magnet’s physical envelope.

The ^1^H Larmor frequency, determined by the magnetic field strength, reduced from 23.42 MHz at 21 °C to 22.25 MHz at 150 °C. Thanks to the low thermal coefficient of SmCo (below13 ppm/°C) and a large thermal mass of the setup, drifts of *B*_0_ were kept well below 100 Hz during any single experiment through the entire temperature range. In conjunction with the HTHP probe and the magnet, we developed a fully integrated NMR spectrometer.

**Figure 3:**
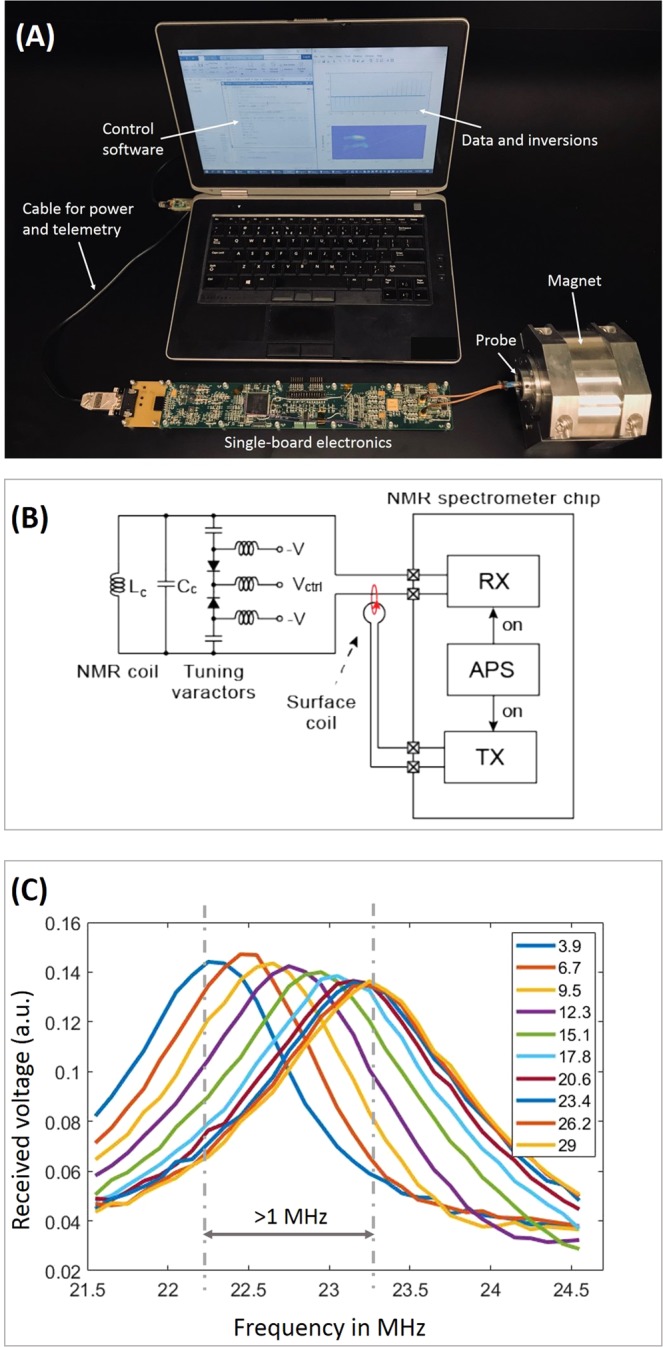
(**A**) A photo of the miniaturized NMR system that includes the magnet, the HTHP probe, the single-board electronics, and a laptop. The power is drawn from the laptop through the USB cable, which also transmits data and commands; (**B**) The schematic of circuit configuration with a small surface coil that injects RF energy into the NMR antenna for circuit tuning; (**C**) The amplitude of received signal at the on-chip receiver as a function of sweeping frequency for different reverse-biased voltage. This experiment was carried out under ambient conditions.

The whole system is shown in Fig. 3A, where the single-board (50 mm by 250 mm) electronics is interfaced to a laptop through a USB cable for both power and data communication. The NMR ASIC is the crucial component that is composed of an RF transmitter (TX), an RF receiver (RX), and an arbitrary pulse sequencer (APS)^6^. The chipset executes pulse programs by delivering RF pulse trains to and acquiring signals from the NMR antenna; a frequency synthesizer, including a PLL (HMC832 by Analog Device) and a quartz oscillator (PX570 by Vectron), provides the radiofrequency reference; and a microcontroller (TMS320F28335PTPQ by Texas Instruments) synthesizes the pulse programs, preprocesses acquired data, and relays the data to the laptop.

Other components include a DAC and a varactor network for circuit tuning, two switches for RF isolation, ADC for signal digitization, power management ICs, and temperature sensors. The spectrometer, with a total power budget of 2 W, can deliver up to 300 mW in RF transmission. More information of the board architecture and operating principles may be found in the Supplementary Materials.

When temperature drifts widely, it is important to match circuit resonance to the proton Larmor frequency, *f*_*L*_. Circuit tuning is achieved by varying capacitance of the varactor arrays (BB639C by Infineon Technologies) in Fig. 3B, where resonance frequency is given by ${f}_{c}=1/(2\pi \sqrt{({C}_{c}+{C}_{v}){L}_{c}})$ with *L*_*c*_ the coil inductance, *C*_*c*_ = 160 pF a fixed tuning capacitor, and *C*_*v*_ the varactor capacitance.

During circuit tuning, a small surface coil injects a fraction of transmitted energy into the NMR antenna, and the amplified signal is detected by the on-chip receiver. By monitoring the signal amplitude, we could determine conditions of circuit resonance. As shown in Fig. 3C, we performed an experiment under ambient conditions on recording the antenna response as a function of sweeping input frequency. As the applied reverse bias increased from 3.9 V to 29 V, *f*_*c*_ shifted by more than 1 MHz. In practice, the circuit response needs to be characterized and tabulated for realtime adjustments.

## HTHP NMR Relaxometry

To characterize the HTHP probe, we conducted NMR relaxation measurements with a Kea2 spectrometer by Magritek. The probe, together with the SmCo magnet, was placed in a temperature-controlled oven. High-pressure fluid lines were connected to the probe for feeding in samples from a micro-reactor (PN 212340E by HiP Company), while hydraulic pressures were regulated by a syringe pump (D-series by Teledyne).

**Figure 4:**
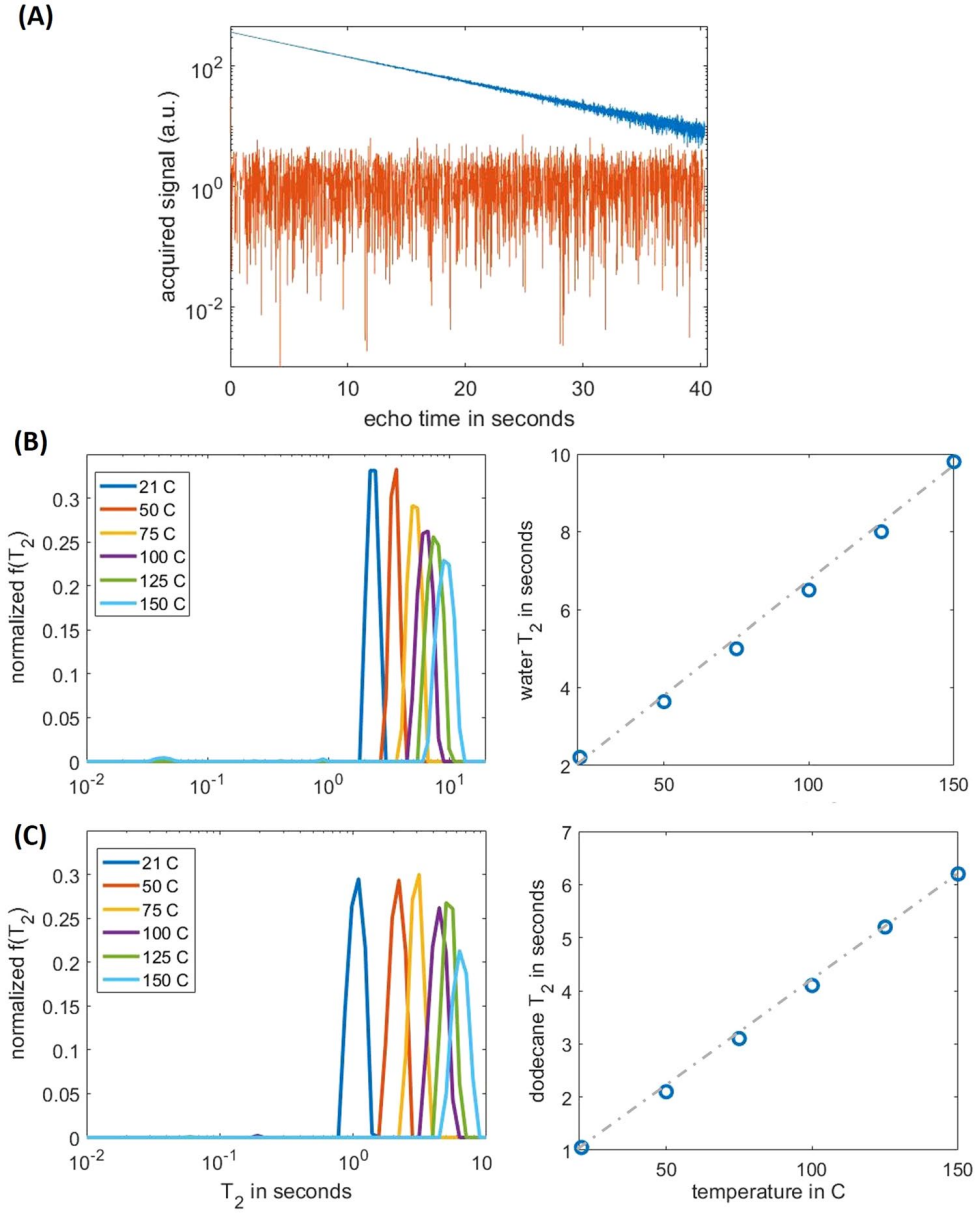
HTHP NMR relaxometry of bulk fluids at different temperatures. (**A**) In-phase (blue) and out-of-phase (red) time-domain relaxation data for water at 150 °C and 137 bar; (**B**) (left) Normalized *T*_2_ spectra of water at different temperatures, and (right) the corresponding log mean; (**C**) (left) Normalized *T*_2_ spectra of dodecane at different temperatures, and (right) the corresponding log mean.

We ran CPMG experiments on two samples, DI water and dodecane, from 21 °C to 150 °C at a constant hydraulic pressure of 137 bar. To measure long *T*_2_’s, it is essential to maintain spin coherence over an extended duration, as shown in Fig. 4A. The Kea2 spectrometer used an oven-controlled oscillator with a ±1 ppb stability. At 23 MHz operating frequency, such stability allows to reliably maintain phase coherence in a CPMG train up to 45 s echo time. Consequently, the system is capable of measuring *T*_2_ up to ca. 10 s.

For both fluids, the temperature-dependent *T*_2_ spectra were clean and narrowly distributed (Fig. 4B,C), consistent with the characteristics of pure bulk fluids. The obtained *T*_2_ at 21 °C were consistent with measurements from samples of much larger volume (10 mL) measured at a commercial spectrometer (RCA by Magritek) with a substantially smaller *B*_0_ gradient, signifying minimal effects of *B*_0_ inhomogeneity in our system. In addition, both samples showed a linear dependence of *T*_2_ over temperature, a hallmark of motion-averaging regime^37^. Specifically, the slope of measured water *T*_2_ agreed with published results in^26^, while the effect of dissolved oxygen^38^ barred a direct comparison^39^ on dodecane. We also note that the spectra appeared somewhat broadened at elevated temperatures, which might be attributed to deteriorated system SNRs.

**Figure 5:**
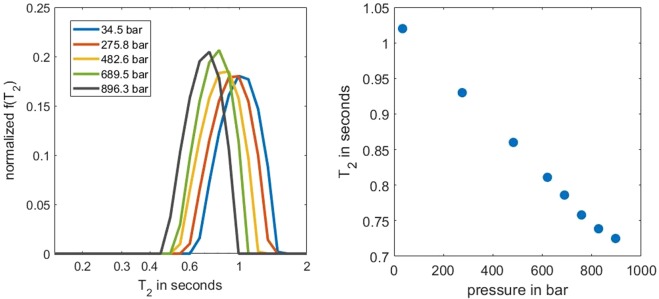
(left) Five selected *T*_2_ spectra of dodecane at 28 °C with varying hydraulic pressures from 34 bar to 896 bar; (right) The log mean of the *T*_2_ spectra as a function of pressure.

We further tested the system by varying hydraulic pressure from 34 bar to 896 bar on a dodecane sample at 28 °C, which resulted in a monotonic diminishing *T*_2_ up to 28%, as shown in Fig. 5. As pressure increased, the more densely packed molecules suppressed their relative motion, and consequently *T*_2_’s decreased^40^. The result agreed with previously reported results^39^, assuming a constant oxygen effect (Supplementary Materials).

## The Miniaturized Spectrometer

Beyond the pure compounds, we benchmarked the fully integrated NMR platform on a suite of complex fluids, both in their chemical composition and spatial configuration, that include food ingredients and multiphasic hydrocarbons. To resolve multiple chemical species and their interactions, we programmed the system to perform 2D *T*_1_–*T*_2_ correlation spectroscopy^41^.

**Figure 6:**
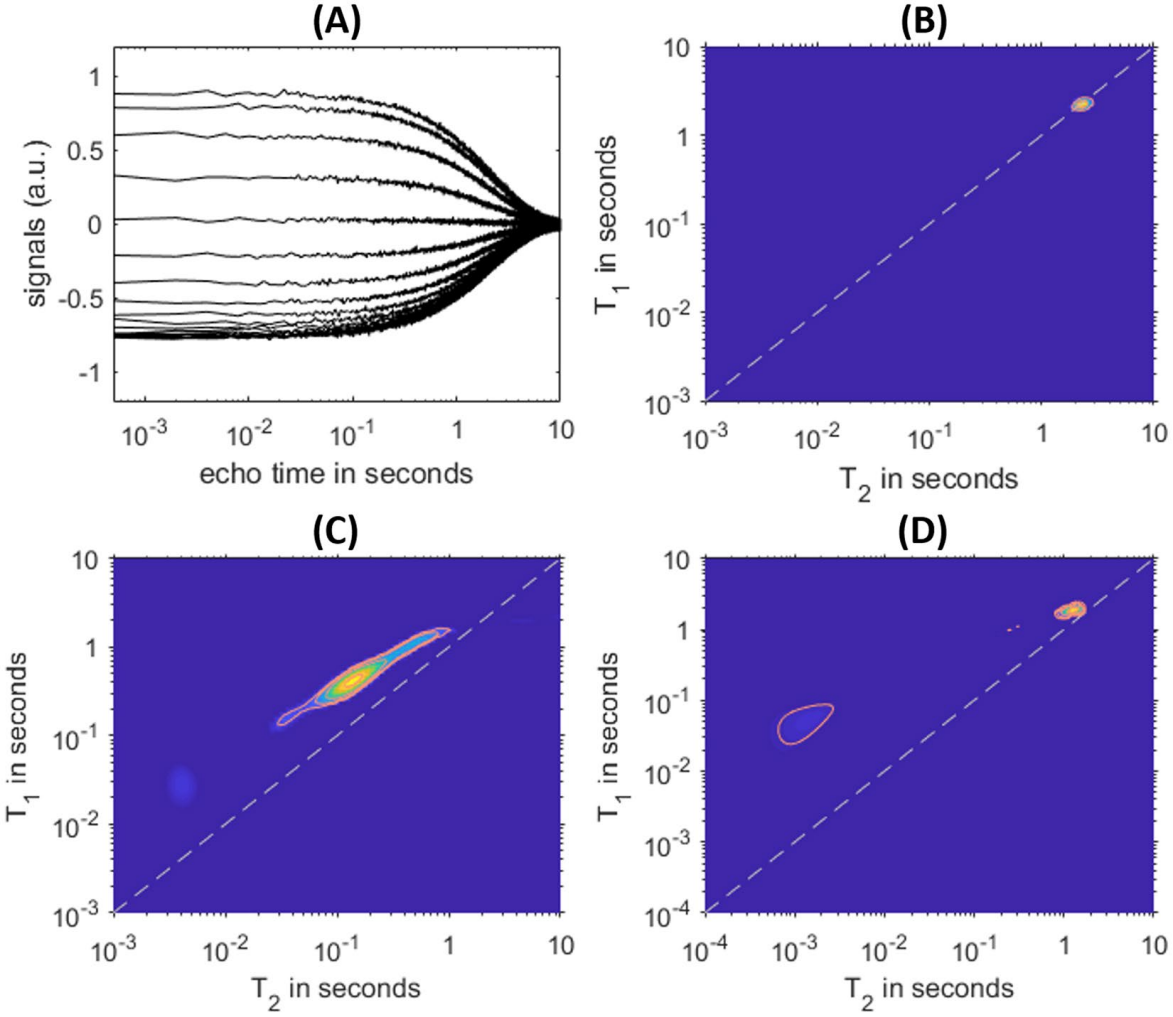
IRCPMG experiments performed by the miniaturized NMR spectrometer on several samples. (**A**) The time-domain signals of a water sample. Each trace is a CPMG train, acquired at different ${\tau }_{1}$ times. *T*_1_–*T*_2_ spectra for water are shown in (**B**), for an egg yolk in (**C**) and for emulsified crude oil with water in (**D**).

As shown in Fig. 3A, a pulse program is initiated in Matlab at the laptop with a set of pulsing and acquisition parameters, which is transmitted to the microprocessor and further relayed to the NMR ASIC for execution. For acquiring a *T*_1_–*T*_2_ spectrum, we used the inversion recovery-CPMG (IRCPMG) sequence with the function^41^:

1 $$S({\tau }_{1},{\tau }_{2})=\int \,(1-2{e}^{-{\tau }_{1}/{T}_{1}}){e}^{-{\tau }_{2}/{T}_{2}}f({T}_{1},{T}_{2})d{T}_{1}d{T}_{2},$$

where ${\tau }_{1}$ and ${\tau }_{2}$ are *T*_1_ encoding and echo time, respectively, with corresponding signals $S({\tau }_{1},{\tau }_{2})$ shown in Fig. 6A. Subsequently, the FLI routine is applied that inverts the time-domain signal to a *T*_1_–*T*_2_ correlation distribution, $f({T}_{1},{T}_{2})$.

We show *T*_1_–*T*_2_ spectra of three fluids with measurements taken at echo spacing = 0.25 ms. Under ambient conditions, the system had a Gaussian noise and a single-scan SNR of ca. 40. In Fig. 6B, the water sample shows a tight peak at ${T}_{1}={T}_{2}=2.2\,{\rm{s}}$, manifesting the unrestricted motion of molecules in their bulk state. In Fig. 6C, a fresh egg yolk shows a spread of distribution along the line of ${T}_{1}/{T}_{2}=2$, reflecting the complex interactions of water, free-floating proteins, and protein-fat aggregates^42,43^. The further elevated *T*_1_/*T*_2_ ratio of the fast-relaxation components at *T*_2_ ~ 4 ms indicates additional motion slowdowns of large molecules^44^.

In Fig. 6D, we performed experiments on a sample of emulsified heavy oil (with density of 0.978 g/cm^3^) with water of 60% volume fraction, a common encounter in the upstream oilfield as well as refinery facilities. The turbid fluid manifested a distinctive oil peak at *T*_2_ ~ 1 ms and a large *T*_1_/*T*_2_ ratio over 100, likely originating from a combination of slow molecular motion and proton-electron interactions by paramagnetic species in asphaltene molecules^45^. Comparing to bulk water, the emulsified water presented a shortened *T*_2_ and a slightly elevated *T*_1_/*T*_2_ ratio, thanks to its interaction with the crude and emulsifiers.

## Discussion

Traditional HTHP NMR is notoriously difficult to perform, given the size and complexity of the HTHP management system. In this regard, reducing the overall sensor footprint helps tremendously alleviate the hardware requirements. Nevertheless, difficulties need to be overcome to achieve the designed functionalities, such as elimination of surface effects, pressure compensation of the probe, and tight control of temperature drift and of overall system stability, including electronics, phase coherence, and sample configurations.

In this work, geometry of the NMR probe is optimized for minimizing frictions in introducing and discharging samples. This “flowline” design is ideal for applications where frequent fluid displacements are either desired or required, such as in online monitoring of fluid properties^15^, investigation of chemical reactions with changing reagents^46^, and evaluation of sampled fluids in oil wells^47^.

Although we focus on relaxometry, the sensor assembly can be extended to other types of NMR measurements with relative ease. For example, a pair of small gradient coils may be added for diffusion measurements^48^; shimming coils and electronics may also be included for chemical-shift spectroscopy^6^. Those modalities can be developed separately to further expand the use of portable instruments for HPHT NMR in a wider context.

We also note that PEEK polymer has its limits. For example, it has a glass transition temperature of 143 °C and therefore could fail at temperatures well above *T*_*g*_; It is relatively weak and requires pressure compensation for HP applications, which complicates the probe design. Alternative materials, such as sapphire^49^, zirconia^50^, and diamond^51^, do exist and merit considerations.

## Supplementary information

Supplementary info
