# Supplementary material for: A miniaturized spectrometer for NMR relaxometry under extreme conditions
Source: Sci Rep. 2019 Aug 1;9:11174. doi: 10.1038/s41598-019-47634-2 (PMC6673705; doi:10.1038/s41598-019-47634-2)
Supplement: Supplementary file 1 — Supplementary info [file 41598_2019_47634_MOESM1_ESM.pdf]

# Supplementary information

## A miniaturized spectrometer for NMR relaxometry under extreme conditions

Yiqiao Tang[1*], David McCowan[1], Yi-Qiao Song[1]

[1] *Schlumberger-Doll Research, Cambridge, MA 02139*

### The miniaturized NMR spectrometer

The architecture and interfaces of the single-board NMR spectrometer are shown in Fig. S1. At the heart of the miniaturized electronics is a 48-pin ASIC chip that includes a receiver (RX), a transmitter (TX), and an arbitrary pulse sequencer. Full details of the chip design and characterization may be found in reference [1]. In short, the chipset executes a prescribed pulse program that was constructed at the laptop and relayed through the microcontroller. Moreover, the microcontroller starts an experiment by enabling the "EN" pin, provides the clock through SPI for sequence timing, sets the exact operating RF at the RF synthesizer, and preprocesses the acquired data, which is subsequently transmitted to the laptop. The laptop is also used for constructing the pulse sequence, supplying power to the spectrometer, and performing inversions. The overall board layout and part placement are shown in Fig. S2. Software to control the device was written in C/C++ on the microcontroller and in Matlab on the laptop.

In the current design, two RF switches, one after TX (TX_SW) and one before RX (RX_SW),

are used to facilitate both standard measurements and circuit tuning. The status of switches is determined via logic signals from both microcontroller and ASIC outputs. In an NMR measurement, TX_SW and RX_SW are opened and closed sequentially for RF isolation, e.g. during pulsing, TX_SW is closed while RX_SW is open; and during acquisition, TX_SW is open while RX_SW is closed. In contrast, in circuit tuning TX_SW is open while RX_SW is closed.

The goal of circuit tuning is to match circuit resonance to Larmor frequency of hydrogen nuclei. To change the circuit resonance, we utilize a capacitor network that consists of eight varactors (BB639C by Infineon Technologies) in parallel. The network can be tuned by varying the applied reverse bias that continuously outputs capacitance from 2.5 pF to 21.5 pF. On resonance, the circuit most effectively amplifies the small RF energy from ASIC TX, which is injected by inductive coupling through a surface coil, and thereby corresponds to a maximal signal at ASIC RX. A demonstration of changing circuit resonance at different bias voltage is shown in Fig. 3C.

**Oxygen effect on relaxation spectra of dodecane**

Dissolved oxygen is paramagnetic and could shorten measured relaxation times of fluids. We approximate the oxygen effect by an additional relaxation time, $T_{2,ox}$, and accordingly the measured $T_2$, $T_{2,mea}$, becomes [2]:

$$\frac{1}{T_{2,mea}} = \frac{1}{T_{2,ox}} + \frac{1}{T_2}, \tag{1}$$

where $T_2$ is the relaxation time of the fluid. In Fig. S3, we plot $T_{2,mea}$ as a function of temperature and pressure, in juxtaposition to $T_{1,mea}$ of deoxygenated dodecane reported in [3] at a different Larmor frequency (90 MHz). Assuming oxygen effect followed the same trend on both $T_1$ and $T_2$ over the external variables, we deduced that the effect was constant in the isothermal process, while diminished in the isobaric process.

### $\mathbf{T_2}$ spectra of dodecane in a machined PEEK capillary

Here, we show an example of using a bad capillary tube. The starting material was a glass-filed PEEK shaft, which was drilled through to create the capillary. A similar construct shown in Fig. 2A was machined and subsequently placed in the HTHP vessel. Relaxation measurements were taken at temperatures from 21 °C to 150 °C at 10 bar. Rather than narrowly distributed $T_2$'s, we saw a minor fast-relaxation component around 100 ms at 21 °C. This component progressively grew as a function of increasing temperature and completely obscured the bulk fluid signals over 75 °C. Such capillary construct is unfit for measurements at elevated temperatures.


1. Ha, D., Paulsen, J. L., Sun, N., Song, Y.-Q. & Ham, D. Scalable NMR spectroscopy with semiconductor chips. *Proc. Natl. Acad. Sci. U.S.A.* **111**, 11955–11960 (2014).

2. Mutina, A. & Hürlimann, M. Effect of oxygen on the NMR relaxation properties of crude oils. *Applied Magnetic Resonance* **29**, 503 (2005).

3. Zega, J. A. *Spin-lattice relaxation in normal alkanes at elevated pressures*. Ph.D. thesis (1991).

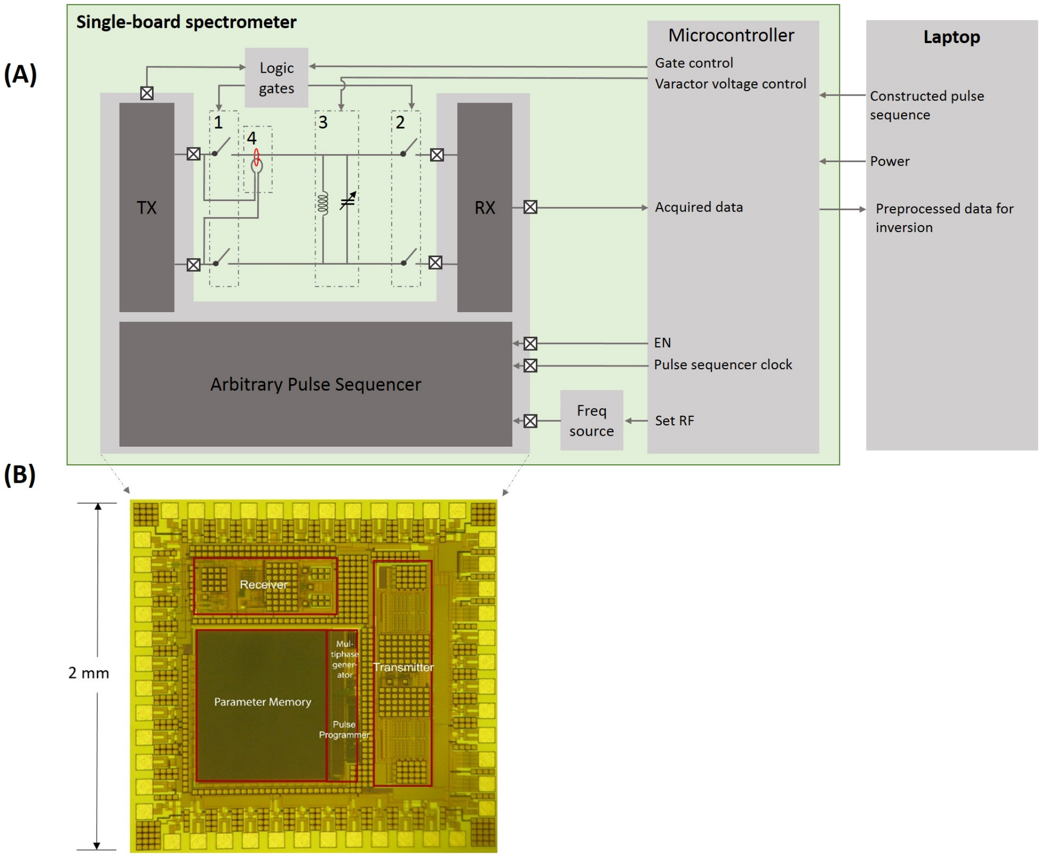


Figure 1: (A) Block diagrams of the ASIC-based NMR spectrometer. The four primary functional units are an ASIC chip (that includes transmitter, TX, receiver, RX, and an arbitrary pulse sequencer), an RF frequency source, an array of logic gates and a microcontroller. RF pulses are transmitted to the resonant circuit, shown in box 3, that consists of an NMR probe and a varactor network. The NMR signal from the probe is received at ASIC RX. The received signals are amplified by a chain of on-chip low-noise amplifiers before relaying to the microcontroller for further processing. Two RF switches, shown in box 1 and 2 for TX and RX respectively, are used to perform both standard NMR measurements and circuit tuning. The surface coil for circuit tuning is highlighted in box 4. The microcontroller also interfaces to the laptop for data communication and operating power. (B) A micrograph of the NMR ASIC chip.

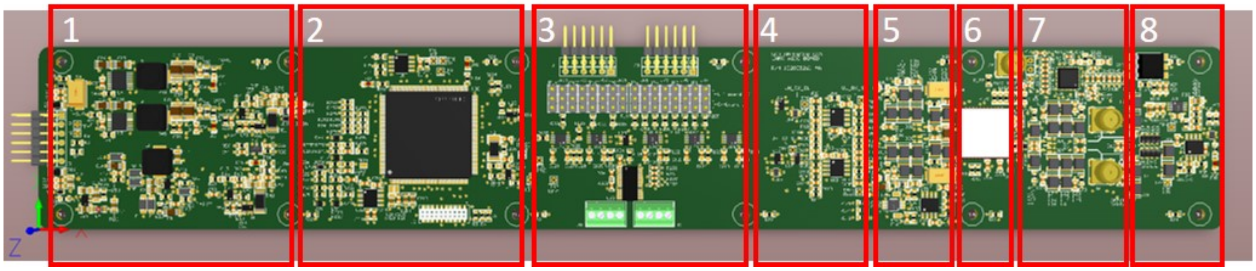


Figure 2: The rendered single-board electronics via Altium software. The primary sections of the integrated board are: 1. Power Supply: all board voltage are generated from a single +5V source; 2. Microcontroller: TMS320F28335PTPQ by TI; 3. External interfaces. The top connectors are for testing and debug. The two small green connectors interface to two resistance temperature detectors; 4. Logic gates to modulate the status of TX and RX switches; 5. TX switch circuit; 6. NMR ASIC IC; 7. RX switch circuit, PLL IC and two SMA connectors that connect to the NMR probe; 8. Varactors, tuning coil and tuning control circuit.

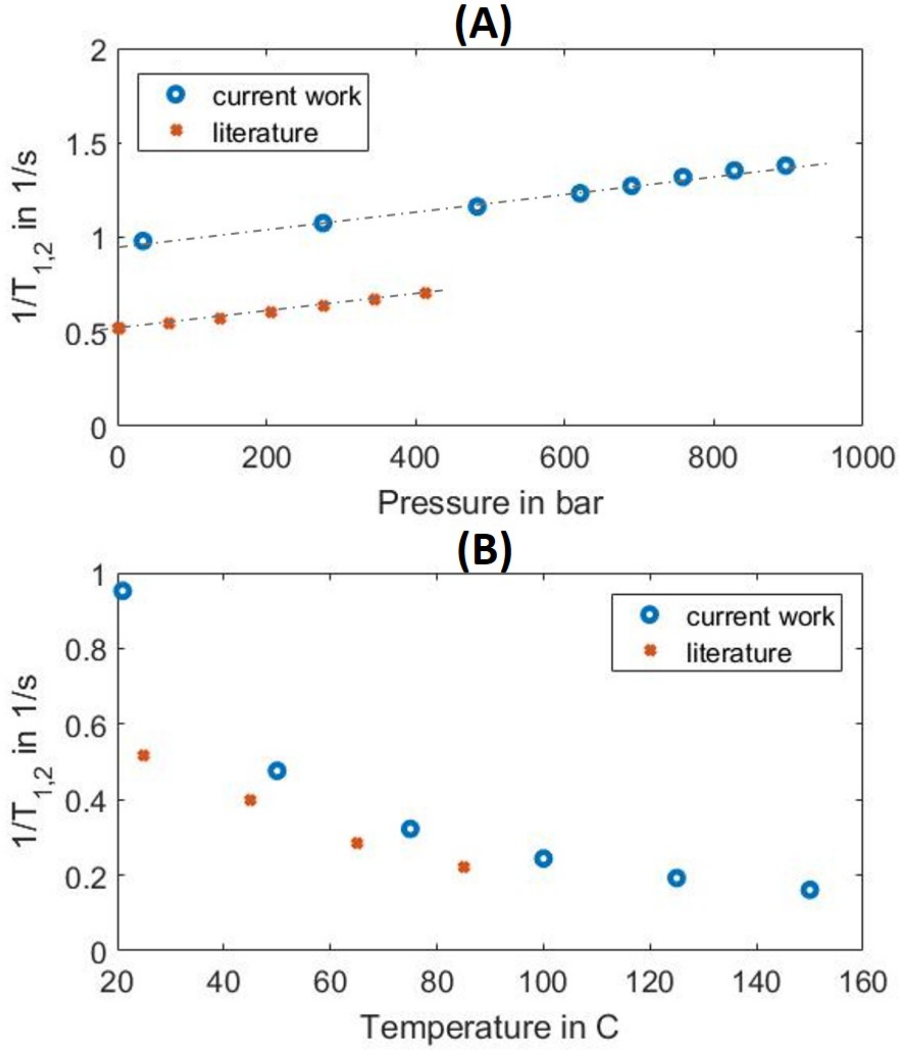


Figure 3: Measured $T_2$ of dodecane in the current work (blue circles) vs $T_1$ of the same, but deoxygenated, fluid reported in [3] (red crosses). (A) Relaxation times as a function of pressures. The two parallel, dotted lines are for visual guide; (B) Relaxation times as a function of temperature.

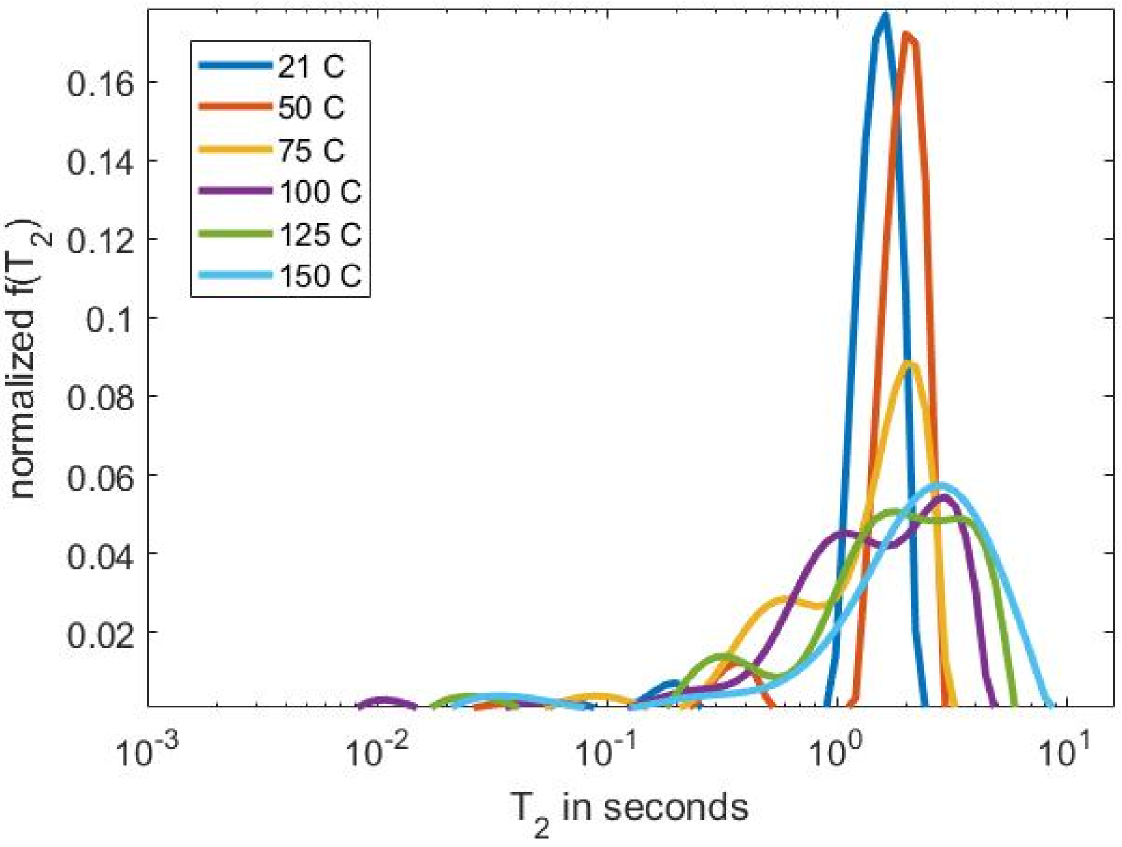


Figure 4: $T_2$ spectra of dodecane in a machined glass-filled PEEK capillary at different temperatures and a constant pressure (10 bar).
